# Metastasis-Associated in Colon Cancer 1 Is a Novel Survival-Related Biomarker for Human Patients with Renal Pelvis Carcinoma

**DOI:** 10.1371/journal.pone.0100161

**Published:** 2014-06-20

**Authors:** Hailong Hu, Dawei Tian, Tao Chen, Ruifa Han, Yan Sun, Changli Wu

**Affiliations:** Department of Urology, Tianjin Institute of Urology, Second Hospital of Tianjin Medical University, Tianjin, China; University of North Carolina School of Medicine, United States of America

## Abstract

Metastasis-associated in colon cancer 1 (MACC1) has recently been identified as a novel independent prognostic indicator for metastasis occurrence, overall survival and cancer-free survival for patients with colon cancer and other solid tumors. In this study, we investigated the role of MACC1 in the development and progression of renal pelvis carcinoma, a form of upper tract urothelial carcinomas. MACC1 protein has been found in the cytoplasm as well as in the nucleus of the transitional epithelial cells of the normal renal pelvis in immunohistochemical (IHC) assays. Quantitative IHC examinations revealed that MACC1 abnormal abundance in cancerous tissues might represent a biological indicator clinically suggestive of tumor malignancy in the renal pelvis. Furthermore, investigation of the association of MACC1 protein levels with clinicopathological parameters in this study has suggested a correlation of MACC1 expression with tumor-node-metastasis stage and histopathological grade of patients with renal pelvis carcinoma, with elevated MACC1 protein levels frequently associated with higher aggressiveness of the disease. Moreover, both disease-free survival and overall survival for the patients in the high MACC1 expression group were significantly lower than those in the low expression group. Multivariate analysis with a Cox proportional-hazards model suggested that MACC1 is indeed an independent prognostic indicator of overall survival and cancer-free survival for patients with renal pelvis carcinoma. Thus, MACC1 may represent a promising prognostic biomarker candidate, as well as a potential therapeutic target for this disease.

## Introduction

As a malignant tumor arising from the transitional epithelium (a highly elastic epithelial tissue consisting of multiple layers of epithelial cells that line the inner surface of the urinary organs), renal transitional cell carcinoma, or renal urothelial carcinoma (UC) represents the fourth most common cancer in the world [1]. Occurring in the renal pelvis and ureter, upper tract urothelial carcinoma (UTUC) is a disease primarily affecting people between the ages of 50 and 75. Pathologically, UTUC is usually more invasive than bladder UC (60% vs. 15%) and is frequently associated with higher malignancy [2], [3], [4]. UTUC at renal pelvis, or renal pelvis carcinoma (RPC) represents approximately 5% to 6% of all renal UCs and are more difficult to diagnose than bladder UC. In fact, the 5-year survival rate of RPC patients is lower than that found in bladder UC as metastasis accounts for about 70% of cancer-specific death in RPC [5]. Notoriously difficult for clinical diagnosis, UTUC represents a tremendous challenge for characterization on radiological imaging, as well as for endoscopic visualization and biopsy. Currently, there are five factors that are the most frequently assessed variables for urothelial carcinoma risk stratification prior to definitive therapy: age, tumor architecture, cytology, biopsy tumor grade, and presence of hydronephrosisand. However, although pathologic predictive factors such as tumor stage, grade, carcinoma in situ, lymphovascular invasion, and lymph node invasion may be more accurate than the other clinical factors to predict disease recurrence and patient survival [6], such information is usually not available before the patient has suffered a significant loss of renal reserve and is less likely to be able to endure aggressive treatments.

In the last 5 years, researchers have gained great insight into the biology and clinical behavior of UTUC. Considerable progress has been made in the identification of molecular prognostic indicators for patients with urothelial carcinoma and may increase risk prediction accuracy. Signaling molecules that are related to cellular processes such as angiogenesis, cell death, cell adhesion, and cell proliferation have been investigated extensively as the potential prognostic indicators in the development and progression of the disease, such as p53, EGFR, survivin, Bcl-2, Ki-67, E-cadherin, hypoxia-inducible factor 1a (HIF1a), telomerase mRNA component, matrix metalloproteinases (MMP-2, MMP-9, TIMP1 and TIMP-2), and more (reviewed in [7], [8]). Identified in a genome-wide analysis as a differentially expressed gene in human colon cancer tissues and metastases, metastasis-associated in colon cancer 1(MACC1) has been suggested as an independent prognostic indicator of metastasis formation and metastasis-free survival for colon carcinoma patients [9]. Detected in a variety of normal tissues, such as intestine, stomach, pituitary gland, kidney, trachea, pancreas, mammary gland, bone marrow, ovary, lung, heart, liver, and B-lymphoblasts, MACC1 is more abundant in the tissues arising from the endoderm (e.g. intestine and stomach) than the tissues originated from mesoderm (e.g. kidney, heart) or ectoderm (e.g. pituitary and mammary gland). Conceivably, MACC1 might play a role in endoderm-derived organogenesis during embryonic development. Originally discovered in colon cancer, MACC1 overexpression has been demonstrated to promote tumor proliferation, invasion, and metastasis in a wide spectrum of solid tumors including gastrointestinal cancers (e.g. colon cancer [9], [10], gastric carcinoma [11]), hepatocellular carcinoma [12], [13], osteosarcoma [14], glioma [15], [16], lung [17], [18], [19], esophageal [20], pancreatic [21], ovarian [22], cervical and breast cancer [23] (reviewed in [24]). Furthermore, examination of MACC1 expression levels in tumor tissues of various clinical stages has revealed that the highest MACC1 expression level has been observed more often in malignant tumor tissues of patients, who frequently demonstrate more unfavorable clinicopathological features including enhanced lymphnode metastasis and metachronously developed distant metastases. In other cases, such as in rarely metastatic human glioma, MACC1 gene expression is more dramatically up-regulated in the tissues of higher malignancy, reflecting symptomatic deterioration of the disease [15]. Elevated MACC1 expression has thus been suggested to serve as an independent prognostic indicator for cancer recurrence, potential exacerbation from benign into malignant tumors, or under certain circumstances, the onset of metastasis. In addition, MACC1 has also emerged as a predictive indicator for recurrence-free survival, and overall survival (OS) of cancer patients in several quantitative studies performed in various solid tumors (reviewed in [24]). Established as a novel prognostic indicator for metastasis in a broad range of solid tumors, MACC1 has also been proposed to be a target candidate for the development of therapeutic strategies for the intervention with tumor progression and metastasis.

In our present study, we investigated the role of MACC1 in the development and progression of human renal pelvis carcinoma. We have demonstrated in immunohistochemical assays the expression pattern of MACC1 protein in human renal pelvis carcinoma tissue specimens as well as in the normal renal pelvis. In addition, we examined the correlation between MACC1 overexpression and clinicopathological parameters (including age, gender, tumor-node-metastasis (TNM) stage and pathological grade), as well as disease-free survival (DFS) and overall survival (OS) in patients with renal pelvis carcinoma. Finally, we also examined by using univariate and multivariate analyses the eligibility of MACC1 as a novel independent prognostic indicator for renal pelvis carcinoma.

## Materials and Methods

### Ethics statement, patients and tissue specimens

A total of 73 RPC patients were recruited for this study during January 2002 and January 2005. The RPC patients participated in this study included 52 males and 21 females. The average age was 61.2 years with a range of 48–79 years. The patients underwent radical nephroureterectomy with complete clinical history records at the Department of Urological Surgery, the Second Hospital of Tianjin Medical University. Patients who had developed metastasis and received post-operative chemotherapy or radiotherapy were excluded from this study. Normal tissue sections in this study were derived from 32 adjacent non-tumorous renal pelvis epithelial tissues (ANRPETs, at least 2 cm distance from the tumor edge) and 34 normal renal pelvis epithelial tissues (NRPETs, urothelial tissues from renal cancer patients, which are less than 3 cm in diameter with intact tissue cell polarity and are considered to be normal epithelial tissues).

All diagnoses of RPCs were confirmed and further classified by postoperative histopathological examination following the criteria of 2004 World Health Organization Classification. Tumor cellular differentiation grades were classified according to the criteria of 1997 World Health Organization Classification. Tumor clinical stage was determined according to the seventh edition of TNM Classification of American Joint Committee on Cancer. Each sample had been fixed in formalin, routinely processed, and embedded in paraffin. Postoperative follow-up data were obtained from all patients, with a median follow-up of 41.5 months (ranging from 8 to 54 months). This study was approved by the Ethics Committee of Second Hospital, Tianjin Medical University, and written informed consent for the use of the specimens from each patient enrolled was obtained accordingly.

### Clinical data collection

All clinical data were prospectively gathered, processed and stored according to the criteria/regulations described above. The clinical variables recorded included gender, age, histological type, TNM stage and nuclear grade (Table 1). Follow-up data were collected at interim physical examinations of the patients. For each patient, disease-free survival (DFS) in this study is defined as the time interval between the date of diagnosis to first recurrence (including local or regional recurrence and systemic metastasis). Overall survival (OS) is defined as the interval between the date of surgery and the date of death. Patients, who were alive at the last follow-up were censored on the date of their last visit to the clinic, and patients, who died from causes other than RPC were censored at the time of their death.

**Table 1:** The average optical density (AOD) of immunohistochemical (IHC) staining strength of MACC1 was measured quantitatively by Image Pro Plus in renal pelvis carcinoma (RPC) tissue specimens, adjacent non-tumorous renal pelvis epithelial tissue (ANRPET) and normal renal pelvis epithelial tissue (NRPET) specimens.

	RPC	Normal epithelium (NE)	
		ANRPETs	NRPETs
Case (MACC1 positive)	73(66)	32(24)	41(29)
MACC1 AOD score	0.0648±0.0312	0.0218±0.0144	0.0210±0.0151
		0.0213±0.0147	
*P* value		0.8454*	
	0.0001#		

Since there was no substantial difference between ANRPETs and NRPETs (*p*>0.05), these two groups were combined as a single group, ‘normal epithelium (NE)’.

*ANRPETs VS NRPETs.

# RPC VS NE.

### Immunohistochemistry (IHC)

The representative paraffin blocks displaying typical tumorous RPC tissues, and normal tissues including ANRPETs and NRPETs were selected for immunohistochemical staining after we carefully examined the hematoxylin–eosin staining slides of all surgical specimens. We performed IHC staining for MACC1 by a two-step peroxidase-conjugated polymer method using Chemmate Envision Detection kit. Briefly, sections were de-waxed with xylene and rehydrated through a graded series of ethanol. The slides were then subjected to heat-induced epitope retrieval using microwave oven in 0.01 M citrate buffer (pH 6.0) for 18 min and were cooled for 30 min at room temperature. The slides were incubated in 3% hydrogen peroxide for 10 min to inactivate the endogenous peroxidase. The sections were then incubated with anti-MACC1 antibody (mouse polyclonal, Ig G, ab106579; 1∶50 diluted, Abcam, Cambridge, UK) as primary antibodies overnight at 4°C in a humidified chamber. After washed in PBS, the sections were incubated with biotinylated rabbit anti-mouse immunoglobulin (Dakopatts, Glostrup, Denmark) diluted at 1∶200 for 30 minutes followed by another 30 minutes’ incubation with peroxidase-conjugated streptavidine (Dakopatts) diluted at 1∶300. Subsequently, the sections were subjected to diaminobenzidine and then counterstained by hematoxylin. Sections from human colon cancer tissues were included as positive controls in each run. The positive controls showed clear positive staining and the intensity was very similar among different runs. The negative control was performed with non-immune bovine serum albumin as a replacement of the primary antibody at the same concentration in each staining run. The sections were washed three times with PBS between each step.

### Quantitative evaluation of immunohistochemical staining

The immunohistochemical staining of MACC1 was quantitatively analyzed by visual assessment and Image Pro-Plus in this study. Visual assessment scoring of MACC1 expression levels was performed by one pathologist followed by reexamination/reconfirmation from another pathologist. The proportion of tumor cells was scored as follows: 0 (no positive tumor cells), 1 (<10% positive tumor cells), 2 (10–50% positive tumor cells), and 3 (>50% positive tumor cells). The intensity of staining was graded according to the following criteria: 0 (no staining), 1 (weak staining  =  light yellow), 2 (moderate staining  =  yellow brown), and 3 (strong staining  =  brown). The staining index was calculated by multiplying the staining intensity score and the proportion of positive tumor cells. Using this method of assessment, we evaluated MACC1 protein expression levels in benign renal pelvis epithelia and malignant lesions by determining the staining index, resulting in scores as 0, 1, 2, 3, 4, 6 or 9. For statistical purposes, IHC scores were grouped into two groups, low expression group (≤4) and high expression group (≥6).

As for the Image Pro-Plus scoring system, MACC1 expression was evaluated quantitatively using the Image Pro Plus 6.0 analysis system (Media Cybernetics, Silver Spring, MD) introduced by Xavier and the results were reexamined by another two pathologists independently after the first round of analysis. Briefly, 10 digital images at 1360×1024 pixel resolution and 400×magnification were captured by the DP 70 CCD camera (Olympus, Japan) coupled to an Olympus AX-70 microscope (Olympus). Quantification of area stained and the integrated optical density (IOD) of MACC1 in each image was determined afterwards and subjected for further analysis. Average optical density (AOD  =  IOD/Area) was used in this study for statistical analysis.

To facilitate statistical analysis for survival data, the entire cohort of patients were divided into two groups, high MACC1 expression group (60.6%) and low MACC1 expression group (39.4%), respectively.

### Statistical analysis

Results were expressed as mean ± standard deviation. Comparison between two groups was carried out using an independent sample *t* test or nonparametric 2-tailed *t* test (Mann–Whitney test). One-way ANOVA (F test) was performed to compare the parameters for multiple groups. Correlation between the expression levels of MACC1 (quantitatively measured according to MACC1 immunohistochemical staining strength) and clinicopathological features in RPC patients was assessed using Pearson's correlation coefficient test. All statistical analyses were performed using SPSS software package (version 17.0, SPSS, Inc., Chicago, IL). 2-sided test was adopted in this study, and *p*<0.05 is considered as statistically significant. The survival rate was estimated using the Kaplan–Meier method. Univariate and multivariate analyses were performed with Cox proportional hazards regression models.

## Results

### Examination of MACC1 protein expression in renal pelvis carcinoma (RPC) tissue, adjacent non-tumorous renal pelvis epithelial tissue (ANRPET) and normal renal pelvis epithelial tissue (NRPET) specimens

**Figure 1:**
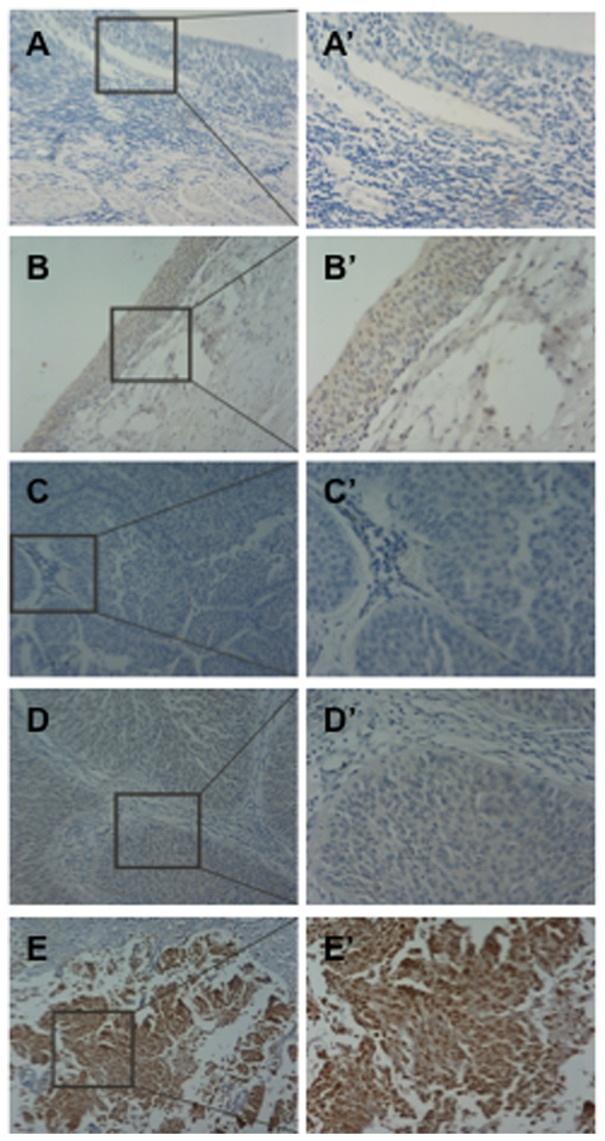
Representative illustrations of immunohistochemical (IHC) staining of MACC1 in the normal renal pelvis (A & A′, B & B′) and in renal pelvis carcinoma (RPC) tissue samples (C–E & C′–E′). (A) negtive control staining; (B) MACC1 positive staining; (C–E) representative images of negtive, low, high MACC1 expression in RPC tissues, respectively. (A′–E′) a higher magnification of the selected area in (A–E), respectively. Magnification: ×100 in (A–E); ×200 in (A′–E′).

Immunohistochemical (IHC) examination of MACC1 protein expression was performed in 73 RPC tissue samples, 32 ANRPET (at least ∼2 cm distance from the tumor edge) and 41 NRPET (urothelial tissues from renal cancer patients, which are less than 3 cm in diameter with intact tissue cell polarity and are considered to be normal epithelial tissues). As revealed in the normal, non-neoplastic renal pelvis tissue sections derived from patients with renal cell carcinoma, MACC1 was stained in the cytoplasm as well as in the nucleus in the epithelial cells residing within the stratified and transitional epithelium (Fig. 1B & B′). In contrast, MACC1 staining was reduced significantly in the stromal tissue. Images in Fig. 1A & A′ are negative control IHC stainings for MACC1.

Positive MACC1 signals were detected in 66 of the 73 RPC cancer lesion samples (∼90.4%), as well as in 24 of the 32 ANRPET (∼75.0%) and 29 of the 41 NRPET (∼70.7%) samples we examined in this study. Images in Fig. 1C–E, 1C′–E′ are the representative MACC1 IHC staining images in RPC tissues with MACC1 detected at negative (Fig. 1C & C′), low (Fig. 1D & D′) and high (Fig. 1E & E′) expression levels, respectively. Although MACC1 expression is mainly present in the cytoplasm in the RPC tissue sections, the nuclear localization of MACC1 is readily discernible as shown in Fig. 1. Interestingly, despite the preferential cytoplasmic staining in the majority of the epithelial cells in the RPC cancerous tissue sections, there are indeed small populations of cells displaying considerably stronger nuclear staining than cytoplasmic staining. In NRPETs, MACC1 was distributed more or less evenly in the cytoplasm and nucleus of the epithelial cells (Fig. 1B & B′). At this stage, we are not clear about the biological consequences of the weaker nuclear staining of MACC1 compared to the cytoplasmic staining exhibited in the malignant RPC tissues.

Next, the immunohistochemical staining strength of MACC1 was measured quantitatively by Image Pro Plus evaluation. Quantification of the stained area, the integrated optical density (IOD) and the average optical density (AOD = IOD/Area) of MACC1 in each image was conducted and subjected for further analysis. As shown in Table 1, the AOD reading of MACC1 was shown as 0.0648±0.0312 in RPC tissues, 0.0218±0.0144 in ANRPETs, and 0.0210±0.0151 in NRPETs, respectively. Since there was no substantial difference between ANRPETs and NRPETs (*p*>0.05), these two groups were combined as a single group, ‘normal epithelium’, as shown in the subsequent statistical analysis we addressed below. As a result, the AOD score of the normal epithelium was 0.0213±0.0147. Collectively, when compared to RPC tissues, the AOD score of immunohistochemically stained normal epithelial tissues was significantly lower, approximating one third of the staining strength identified in RPC (*p*<0.05) (Table 1). These results suggest that MACC1 abnormal abundance might represent a biological indicator clinically suggestive of tumor malignancy in renal pelvis tissues.

### The correlation between MACC1 protein expression levels and prognostic factors in patients with renal pelvis carcinoma

To determine the clinical significance of MACC1 overexpression in the development and progression of RPC, the correlation between MACC1 protein expression levels and the prognostic factors (including age, gender, TNM stage and nuclear grade) in the RPC patients was further investigated (Table 2). Quantitative analysis of IHC staining by Image Pro Plus analysis was performed. As shown in Table 2, elevated MACC1 protein levels appeared to be associated with higher aggressiveness of RPC, with the AOD score of MACC1 in TNM stage Ta, I, II, and III-IV revealed as 0.0355±0.0247, 0.0563±0.0281, 0.0752±0.0264, and 0.0965±0.0185, respectively. Noticeably, MACC1 demonstrated increased abundance in late stage tumor tissues such as TNM stages III–IV, when compared with that in the early stage and the difference was statistically significant (*p*<0.05). In addition, the AOD score of MACC1 in RPC stage Ta was also substantially higher than that measured in the normal epithelium (0.0213±0.0147) (*p*<0.05). As regard to tumor nuclear grade, there was also a statistically significant difference between the AOD scores of MACC1 measured in the low-grade (G1, G2: 0.0509±0.0281) and the scores measured in the high-grade RPC tissues (G3: 0.0729±0.0304) (*p*<0.05). Indeed, there was ∼43.2% increase detected in tumor tissues of grade 3 when compared to grade 1 or 2, suggesting that MACC1 protein abundance may be strongly correlated with the development and progression of RPC.

**Table 2:** The correlation between immunohistochemical scores of MACC1 protein expression (indicated as the values of the average optical density (AOD) measured by Image Pro Plus in renal pelvis carcinoma tissue sections) and prognostic factors (gender, age, TNM stage, and nuclear grade).

Prognostic factors	Case	MACC1		*P*
	number	AOD score		Value
**Gender**				
Male	52	0.0662±0.0305		0.5396
Female	21	0.0612±0.0335		
**Age**				
<61	26	0.0628±0.0343		0.7362
>61	47	0.0654±0.0298		
**TNM stage**				
Ta	8	0.0355±0.0247	0.0526±0.0284	0.0001
T I	37	0.0563±0.0281		
T II	16	0.0752±0.0264	0.0843±0.0253	
T III–IV	12	0.0965±0.0185		
**Nuclear grade**				
G1-2	46	0.0509±0.0281		0.0025
G3	27	0.0729±0.0304		

In contrast, although MACC1 exhibited an increased protein level in the group of older patients (>61 years old) with the AOD score measured as 0.0654±0.0298 when compared to the group of younger patients (<61 years old) (AOD score: 0.0628±0.0343), no statistically significant difference was observed between these two groups (*p*>0.05). Similarly, no statistically significant difference was observed between the male group (AOD score: 0.0662±0.0305) and the female group (AOD score: 0.0612±0.0335) (*p*>0.05) (Table 2). Furthermore, no statistically significant correlation was revealed between MACC1 protein expression and urine cytology or smoking behavior (data not shown).

### The association between MACC1 protein expression levels and overall survival (OS), disease-free survival (DFS) in patients with renal pelvis carcinoma

**Figure 2:**
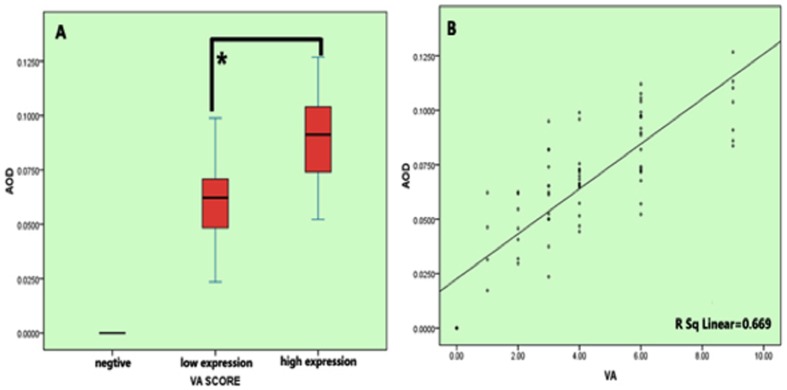
The relationship between the average optical density (AOD) scores of MACC1 immunohistochemical (IHC) staining obtained in Image Pro Plus anaysis and the staining intensity scores determined by visual assessment. (A), Patients with RPC were manually divided into three groups according to their visual assessment scores in IHC stained tissue sections (negative, low MACC1 expression group, and high MACC1 expression group, respectively). The AOD value of MACC1 obtained from Image Pro Plus analysis was evidently increased in the low MACC1 expression group compared to the negative group, and was further elevated in the high MACC1 expression group (*p*<0.001). AOD =  the integrated optical density (IOD)/Area of positive MACC1 staining in each IHC staining image; B, Spearman correlation coefficient analysis measuring the strength of the linear relationship between MACC1 AOD Image Pro Plus scores and visual assessment scores. A statistically significant positive linear relationship was observed between these two sets of scores in RPC (*r* = 0.669, *p*<0.001).

To facilitate statistical analysis, RPC patients were divided into three groups according to the visual assessment of the immunohistochemical staining strength in tissue sections we examined (negative, low MACC1 expression group, and high MACC1 expression group, respectively). The AOD score of MACC1 obtained from Image Pro Plus analysis was evidently increased in the low MACC1 expression group compared to the negative group, and was further elevated in the high MACC1 expression group (*p*<0.001, Fig. 2A). Furthermore, in Spearman correlation coefficient analysis measuring the strength of the linear relationship between MACC1 AOD scores from Image Pro Plus analysis and visual assessment scores, a statistically significant positive linear relationship was observed between these two sets of scores in patients with RPC (*r* = 0.669, *p*<0.001) (Fig. 2B). Thus, we concluded the MACC1 expression pattern deduced from visual assessment indeed faithfully resembles that extracted from the analysis using the Image Pro Plus AOD scoring system.

**Figure 3:**
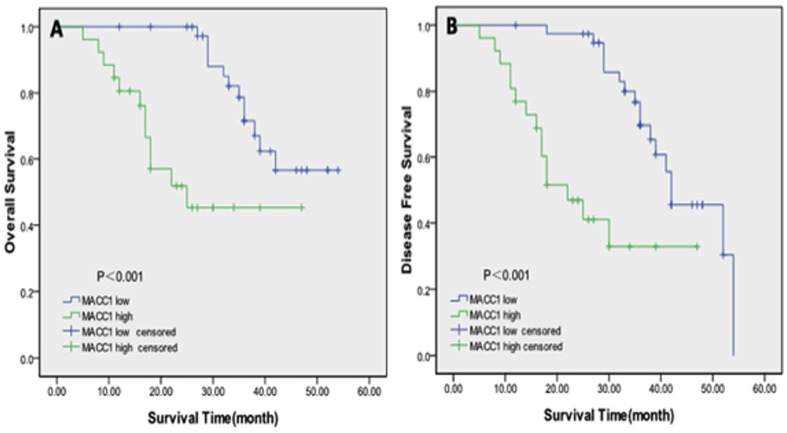
Kaplan–Meier survival analysis. The Kaplan–Meier survival curves revealed that patients in the high MACC1 expression group demonstrated significantly shorter overall survival (OS) (*p*<0.001) (A) and disease-free survival (DFS) (*p*<0.001) (B) rates than those in the low MACC1 expression group.

Next, we examined the prognostic significance of MACC1 overexpression in overall survival (OS) and disease-free survival (DFS) among RPC patients. 28 patients died in the 5-year follow-up, and among the 28 deceased, 18 died with cancer metastasis or local recurrence. As shown in Fig. 3A & B, patients with high MACC1 expression level determined by visual assessment scoring system showed a significantly shorter OS (*p*<0.001) and DFS (*p*<0.001) than the group of patients with low MACC1 expression level. As illustrated in Fig. 3A, in the high MACC1 expression group, the mean OS rate was 29.9 months with the 5-year OS rate approximating 70%. For patients in the low MACC1 expression group, the mean OS rate was 45.6 months, ∼50% higher than the high MACC1 group. In addition, the DFS rate was ∼22 months as revealed in the high MACC1 group, whereas the rate almost doubled in the low MACC1 group (∼42 months) (Fig. 3B). Similarly, the 5-year DFS rate in the low MACC1 group was evidently higher than the rate in the high MACC1 group (57.5% versus 42.3%). Conclusively, high MACC1 expression level is significantly correlated with decreased overall survival and disease-free survival in RPC patients as revealed in the quantitative immunohistochemical analyses.

### Univariate and multivariate analyses established MACC1 as a novel independent prognostic indicator for RPC

In this study, univariate and multivariate analyses were performed for the evaluation of the prognostic power of MACC1 overexpression (Tables 3 & 4). As shown in Table 4, multivariate Cox regression analysis demonstrated that MACC1 expression levels and nuclear grade are indeed independent prognostic indicators for the overall survival of patients with RPC after the adjustment for age, gender and TNM stage (Grade, *p* = 0.001, Hazard ratio: 0.086, 95% CI: 0.019–0.387; MACC1, *p* = 0.024, Hazard ratio: 0.282, 95% CI: 0.094–0.844. Table 4). In addition, MACC1 expression levels and nuclear grade were also identified as independent prognostic factors for cancer-free survival in patients with RPC (Grade, *p* = 0.001, Hazard ratio: 5.444, 95% CI: 1.964–15.093; MACC1, *p* = 0.002, Hazard ratio: 4.207, 95% CI: 1.668–10.613. Table 4).

**Table 3:** Univariate Cox regression analysis of several prognostic factors (age, gender, TNM stage, nuclear grade, and MACC1 protein expression levels) with respect to five-year overall survival (OS) and disease-free survival (DFS) of patients with renal pelvis carcinoma.

Prognostic factors	Overall survival (OS)		Disease-free survival (DFS)	
	HR (95% CI)	*P*	HR (95% CI)	*P*
Age	1.342 (0.59–3.036)	0.479	0.85 (0.411–1.776)	0.674
Gender	1.575 (0.588–4.223)	0.366	0.74 (0.318–1.730)	0.488
Stage	0.335 (0.129–0.869)	0.025	2.153 (0.861–5.384)	0.101
Grade	0.073 (0.017–0.319)	0.001	6.606 (2.459–17.746)	0.001
MACC1 expression	0.226 (0.097–0.529)	0.001	4.420 (2.064–9.464	0.001

‘CI’ refers to confidence interval; ‘HR’ refers to hazard ratio.

**Table 4:** Multivariate Cox regression analysis of several prognostic factors (TNM stage, nuclear grade, and MACC1 protein expression levels) with respect to five-year overall survival (OS) and disease-free survival (DFS) of patients with renal pelvis carcinoma (RPC).

Prognostic factors	Overall survival (OS)		Disease-free survival (DFS)	
	HR (95% CI)	*P*	HR (95% CI)	*P*
Stage	1.246 (0.365–4.251)	0.726	0.524 (0.169–1.624)	0.263
Grade	0.086 (0.019–0.387)	0.001	5.444 (1.964–15.093)	0.001
MACC1 expression	0.282 (0.094–0.844)	0.024	4.207 (1.668–10.613)	0.002

‘CI’ refers to confidence interval; ‘HR’ refers to hazard ratio.

## Discussion

Although renal pelvis carcinoma is relatively less common, it is more difficult to diagnose than bladder urothelial carcinoma and represents an aggressive disease with high recurrence and progression rates. In addition to tobacco smoking, examination of other environmental risk factors has suggested that occupational exposure to industrial chemical substances could also contribute to the development of urothelial carcinoma, e.g. chemotherapy (most notably, cyclophosphamide) and radiation therapy, chronic bladder irritation and infections, as well as arsenic exposure [25]. China has the world's largest smoking population - ∼350 million, approximately one fourth to one third of the world's population of smokers. In addition, as a result of rapid, unprecedented industrial growth, the industrial chemical pollution has also become an alarmingly increasing threat to the health of many people in China in the last 2–3 decades. Chemicals that have been suggested to promote upper tract urethral carcinoma include aromatic amines, such as benzidine (printers), 2-napthylamine (rubber and dye workers), 4-aminobiphenyl (hairdressers), polycyclic aromatic hydrocarbons, such as Benzo(a)pyrene (aluminum workers) and diesel fumes [26]. Furthermore, with improved cancer screening accuracy in China in recent years, the dramatic rise in cancer prevalence in the population has represented a huge challenge in the last 5–10 years. Indeed, a higher frequency of renal pelvis carcinoma has been identified in the last 2–3 decades in China. It is thus important to develop more effective prognostic indicators, and identify high-risk patients for appropriate and timely intervention.

Emerging as a potentially less invasive measurement, assessment of the status of tissue-specific biomarkers is suggested to have the capacity to improve diagnostic and prognostic accuracy in the decision-making process when integrated into current prognostic models. In recent years, biomarkers associated with the prognosis of patients with upper tract urothelial carcinomas have been extensively explored. Indeed, a wide range of tissue-based biomarkers have been reported and evaluated for their potential application as urothelial carcinomas prognostic indicators, such as molecules that regulate cell cycle (e.g. p53, p21, pRb, p27, cyclin D1 and cyclin E1), apoptosis (e.g. survivin, Bcl-2, osteopontin, bax, caspase-3), cell growth and signal transduction (e.g. HER-2, NF-kB, EGFR, beta-catenin, galectin-3, RAS-MAPK), cell migration and invasion (e.g. MMPs), and angiogenesis (e.g. vasohibin-1, VEGF, TSP-1) (reviewed in [7], [8]). As shown in this study, we demonstrated for the first time that an abnormally high level of MACC1 expression is associated with a poor prognosis of renal pelvis carcinoma and is indeed an independent prognostic indicator for disease-free survival and overall survival of patients. Since at this stage we have no effective treatment for patients with metastatic renal pelvis carcinoma, the detection of high levels of MACC1 might help to predict the progression and the prognosis of the disease. Noticeably, in metastatic solid tumor tissues as revealed in previous studies, in addition to increased intensity of MACC1 staining in the cytoplasma, additional nuclear staining has also been recognized suggesting enhanced nuclear translocation and the subsequent elevation of MACC1 transcriptional activities [15], [27], [28]. Indeed, MACC1 translocation from the cytoplasm to the nucleus has been indicated to be intimately associated with metastasis in colon cancer cells [27] and may very well indicate the higher malignancy in the case of glioma cells [15]. In this study, we have observed an even nucleocytoplasmic distribution pattern of intracellular MACC1 protein in the normal renal pelvis epithelium. Surprisingly, in the malignant renal pelvis carcinoma tissues, MACC1 is stained predominantly in the cytoplasma although the expression level of MACC1 is substantially higher than that found in the normal renal pelvis epithelium. Despite the preferential cytoplasmic staining in the majority of the epithelial cells in the renal pelvis carcinoma tissues, there are indeed small populations of cells exhibiting significantly stronger nuclear staining when compared to cytoplasmic staining. Since increased nuclear accumulation of a transcriptional factor usually indicates up-regulated transcriptional activities, we postulate here that those cells demonstrating strikingly stronger MACC1 nuclear staining might represent cancer stem cells, the highly tumorigenic cells that are responsible for tumor initiation and maintenance. It would be interesting in the future to explore the downstream signaling events responsible for MACC1-mediated tumorigenesis in urothelial carcinoma.

The MACC1 gene is located on the minus strand of human chromosome 7p21.1 [9]. As it is well known that chromosomal aneuploidy has been observed frequently in carcinomas, it is not surprising that abnormal amplification, rearrangement of chromosome 7, or gain of the p-arm (particularly 7p21), has been discovered in a wide spectrum of cancers, such as gastric, intestinal, colorectal, pancreatic cancers and astrocytic tumors [19] [9], [29]–[40] (also reviewed in [29]). Indeed, there are many lines of evidence indicating that the pathological amplification or rearrangement of chromosome 7p may very well underlie the increased abundance of MACC1 mRNA and is intimately associated with a concomitant overexpression of the protein throughout tumor growth, invasion, metastasis, and tumor recurrence [9] [27] [30] [41], [42] (also reviewed in [29]). It would certainly be of great interest in the near future to examine whether there is indeed a high level of aberrant genomic DNA amplification and/or rearrangement in the genomic DNA fragment containing the MACC1 locus in patients with renal pelvis carcinoma.

Finally, in the development of blood- or urine-based markers for upper tract urothelial carcinoma, C-reactive protein, Cytokeratin 19 fragment (CYFRA21-1), and humoral factors (alkaline phosphatase and white blood cell counts) have been identified to be able to independently predict overall survival in cancer patients [43], [44], [45]. Interestingly, in a previous study, a high level of MACC1 protein in the serum collected from pancreatic cancer patients has been detected and suggested to be correlated with lymph node metastasis, distant metastasis and a later TNM stage [21]. In addition, examination of the MACC1 transcript level in the blood samples collected from colorectal cancer patients has revealed an abnormal increase of MACC1 transcripts with the highest MACC1 transcript levels identified in individuals with metastases [46]. Further investigation of the correlation of circulating MACC1 transcripts in the plasma with patient survival suggested that high MACC1 levels are associated with unfavorable survival. Currently, we are investigating the possibility of quantification of MACC1 transcripts and proteins in the plasma isolated from patients with renal pelvis carcinoma. Eventually, we would like to investigate the relationship between MACC1 transcript/protein levels in the plasma and the clinical stage of renal pelvis carcinoma, and more importantly, to explore whether MACC1 transcript/protein levels in the plasma would serve as a potential prognostic indicator for the disease.
